# In vivo CRISPR/LbCas12a-mediated knock-in and knock-out in Atlantic salmon (*Salmo salar* L.)

**DOI:** 10.1007/s11248-023-00368-4

**Published:** 2023-09-21

**Authors:** Mari Raudstein, Erik Kjærner-Semb, Morten Barvik, Silje Broll, Anne Hege Straume, Rolf Brudvik Edvardsen

**Affiliations:** Institute of Marine Research, Bergen, Norway

**Keywords:** Aquaculture, Genome editing, HDR, New breeding technologies, Cpf1

## Abstract

Genome editing using the CRISPR/Cas system offers the potential to enhance current breeding programs and introduce desirable genetic traits, including disease resistance, in salmon aquaculture. Several nucleases are available using this system, displaying differences regarding structure, cleavage, and PAM requirement. Cas9 is well established in Atlantic salmon, but Cas12a has yet to be tested in vivo in this species. In the present work, we microinjected salmon embryos with LbCas12a ribonucleoprotein complexes targeting the pigmentation gene *solute carrier family 45 member 2* (*slc45a2*). Using CRISPR/LbCas12a, we were able to knock-out *slc45a2* and knock-in a FLAG sequence element by providing single-stranded DNA templates. High-throughput sequencing revealed perfect HDR rates up to 34.3% and 54.9% in individual larvae using either target or non-target strand template design, respectively. In this work, we demonstrate the in vivo application of CRISPR/LbCas12a in Atlantic salmon, expanding the toolbox for editing the genome of this important aquaculture species.

**Supplementary Information:**

The online version contains supplementary material available at 10.1007/s11248-023-00368-4.

## Introduction

Norway is the world’s largest producer of farmed Atlantic salmon (*Salmo salar*) and exported over 1 million tons of fish in 2022. The salmon industry has steadily increased over the years, but further expansion is currently hindered due to several sustainability issues. One of the problems is infectious diseases attributable to viruses, bacteria, or parasites (Sommerset et al. 2023). To combat this problem, breeding programs have been applied to develop fish with more robust performance in the sea pens (Thodesen and Gjedrem 2006; Kjøglum et al. 2008). However, selective breeding is time-consuming, especially in species with a long generation time like the Atlantic salmon. New breeding technologies such as genome editing (GE) using the CRISPR/Cas (Clustered Regularly Interspaced Short Palindromic Repeats/CRISPR-associated) system may facilitate current breeding programs and further introduce favorable genetic traits including disease resistance (Gratacap et al. 2019) without the need for breeding the fish for many generations.

CRISPR/Cas9 was first utilized as a tool for GE in 2012 by Jennifer Doudna and Emmanuelle Charpentier (Jinek et al. 2012). Since then, it has become widely used owing to its efficiency and versatility. The system consists of two components, the Cas nuclease and a single guide (sg) RNA molecule, together forming the nuclease effector complex. The sgRNA molecule is comprised of both a crisprRNA (crRNA) and a trans-activating crRNA (tracrRNA). The tracrRNA-part enables the recruitment of the nuclease, while the crRNA is programmable and can be designed to target a specific region within the genome. In this manner, the nuclease effector complex is guided to the target site of interest by the pre-programmed crRNA. Prior to binding the DNA, the nuclease effector complex requires the recognition of a short sequence termed the protospacer adjacent motif (PAM). Once bound, the nuclease initiates a double-strand break (DSB) to the DNA, and the endogenous repair mechanisms that follow are exploited to do GE. Repair usually occurs by two main mechanisms: non-homologous end joining (NHEJ) or homology-directed repair (HDR). During NHEJ, the cut ends are trimmed, nucleotides are recruited, and the strands are re-ligated. However, this process often results in erroneous repair with random insertions and deletions (indels), and may lead to a gene knock-out (KO) if produced in a coding exon. This approach allows us to study KO phenotypes and has previously been applied in salmon (Wargelius et al. 2016; Datsomor et al. 2019) as well as other aquaculture species such as common carp (*Cyprinus carpio)* (Zhong et al. 2016), Nile tilapia (*Oreochromis niloticus*) (Jiang et al. 2017), and rainbow trout (*Oncorhynchus mykiss)* (Cleveland et al. 2018). On the other hand, the HDR mechanism can be utilized to knock-in (KI) genetic material by providing a donor template together with the nuclease effector complex. KI by HDR has been done in salmon using oligodeoxynucleotides (ODNs) to perform single nucleotide replacement or insert FLAG sequence elements (Straume et al. 2020, 2021). Inserting larger genetic material remains challenging but has been achieved in other aquaculture species such as channel catfish (*Ictalurus punctatus)* (Simora et al. 2020; Xing et al. 2022).

Cas9 derived from *Streptococcus pyogenes* was among the first nucleases to be established for GE and is still widely used today. However, the restriction for a particular PAM site narrows down the scope of target sites available for GE. More recently, novel nucleases derived from other bacterial species, e.g., Cas12a (previously Cpf1) from *Acidaminococcus* sp. (AsCas12a) or Lachnospiraceae bacterium (LbCas12a), have also been shown effective for GE (Zetsche et al. 2015). The nucleases Cas9 and Cas12a represent different types of Cas enzymes, type II and V, respectively. The two types differ in characteristics such as structure, cleavage, and importantly, PAM requirement. Cas9 forms a complex with the sgRNA, recognizes PAM 5′-NGG-3′ downstream of the protospacer, and usually produces a blunt-end cleavage 3 bases upstream of the PAM (Jinek et al. 2012). In contrast, Cas12a forms a complex with the crRNA only, recognizes PAM 5′-TTTV-3′ (V represents A, C, or G) upstream of the protospacer, and cleaves about 18 bp downstream of the PAM on the non-target strand, and 23 bp downstream on the target strand, producing staggered ends (Zetsche et al. 2015).

Its unique characteristics make Cas12a a promising tool for GE for several reasons. Firstly, the T-rich PAM requirement enables GE in AT-rich regions not accessible to Cas9. Secondly, the shorter crRNA utilized by Cas12a is easier and cheaper to synthesize compared to the crRNA and tracrRNA molecules needed by Cas9 (Zetsche et al. 2015). Finally, Cas12a has been shown to induce higher HDR efficiency than Cas9 in zebrafish (Moreno-Mateos et al. 2017). While Cas12a has been established in model organisms such as zebrafish (Meshalkina et al. 2020; Fernandez et al. 2018; Han et al. 2022) and silkworm (Dong et al. 2020), as well as salmonid cell lines (Gratacap et al. 2020), the in vivo application has yet to be reported in any aquaculture species. Therefore, this study aimed to investigate if the CRISPR/Cas12a system could be established as a GE tool in Atlantic salmon. To address this question, we targeted the pigmentation gene *solute carrier family 45 member 2* (*slc45a2*) and microinjected salmon embryos with LbCas12a ribonucleoprotein complexes (RNPs). We also injected RNPs in combination with a ODN FLAG template to assess the HDR efficiency of LbCas12a versus Cas9. We performed high-throughput sequencing (HTS) of individual larvae to determine the efficiency and accuracy of the integrations. To our knowledge, this is the first report of GE using the CRISPR/LbCas12a system in vivo Atlantic salmon. By implementing the use of LbCas12a, we expand the toolbox for editing the genome of this important aquaculture species.

## Materials and methods

### Target site selection

Target site selection for Cas9 GE of *slc45a2* is described in previous work (Edvardsen et al. 2014). Target site selection for LbCas12a GE was done as follows: the gene sequence of *slc45a2* was obtained from the Atlantic salmon reference genome assembly v2 on the National Center for Biotechnology Information (NCBI) website. (GenBank: GCF_000233375.1, NCBI) (https://www.ncbi.nlm.nih.gov/gene/106563596/: NC_027300.1 (117874712..117899795, complement)). Target sites containing the PAM site 5′-TTTV-3′ for CRISPR/Cas12a cleavage were identified using Geneious Prime software (v. 11.0.12). Candidate target sites were selected from the first exons and BLASTN (Altschul et al. 1990), available on the NCBI website, was used to screen for crRNA sequences with limited chance for off-target cleavage in the Atlantic salmon genome (ICSASG_v2, GCF_000233375.1).

### crRNA and sgRNA preparation

Alt-R L.b. Cas12a crRNAs targeting *slc45a2* exon 1 and exon 2 were ordered from Integrated DNA Technologies (IDT) (Coralville, USA). Cas9 sgRNA targeting *slc45a2* exon 6 was synthesized as described in Gagnon et al. (2014) with the following exceptions: the QIAquick PCR Purification Kit (Qiagen) was used to purify the sgDNA templates, the HiScribe T7 Quick High Yield RNA synthesis kit (NEB) was used for in vitro transcription, and the RNeasy Mini Kit (Qiagen) was used to purify the synthesized sgRNA. An overview of crRNA and sgRNA sequences can be found in Supplementary File 1.

### ODN design

ODN templates for KI using LbCas12a were designed based on previous studies (Straume et al. 2021; Moreno-Mateos et al. 2017; Richardson et al. 2016). One target and one non-target strand template were designed asymmetrically by copying 90/36 nt from each side of the cut sites, with a 27–29 nt insert comprised of the (CG-)FLAG-TAA sequence. The CG addition was included when needed to keep the open reading frame of FLAG, whereas the stop codon TAA was included to ensure an albino phenotype. Finally, the PAM sites were mutated to avoid repeated cutting. ODN design for KI using Cas9 is described previously (Straume et al. 2021). ODN template sequences can be found in Supplementary File 1. ODNs were ordered from IDT (Coralville, USA).

### Cas nucleases

Alt-R L.b. Cas12a (Cpf1) Ultra and Alt-R S.p. Cas9 Nuclease V3 were ordered from IDT (Leuven, Belgium).

### Fertilization

Salmon eggs and sperm were obtained from Mowi (Askøy, Norway). The eggs were fertilized in 0.5 mM reduced glutathione (Sigma-Aldrich) solution (pH 10) to prevent chorion hardening. The embryos were incubated for 3 + hours at 6–8 ℃ until the first cell was visible.

### Ribonucleoprotein complex assembly

Ribonucleoprotein (RNP) complexes were assembled by mixing the appropriate Cas nuclease with crRNA or sgRNA to a final concentration of 100 ng/µL of both components. The RNP complexes were incubated at room temperature for 20 min.

### Microinjection

Glass capillaries (O.D 1.0 mm, I.D 0.50 mm, 10 cm) (Sutter Instrument) were pulled using a PC-100 needle puller (Narishige, Japan). Fertilized eggs were injected using a FemtoJet® 4i injector (Eppendorf). The injection mix contained the pre-formed RNP complex (100 ng/µL), and for the KI experiments, the appropriate ODN template (1.5 µM). Following injection, the eggs were incubated at 6 ℃ until sampling.

### Sampling

The larvae were killed with an overdose of buffered MS-222: Tricaine Methane Sulfonate and sampled after 600–700 day-degrees. Individuals showing albino and mosaic pigmentation phenotypes were selected. A non-injected control was included. DNA was isolated from fin clips using the Agencourt DNAdvance Kit (Beckman Coulter) according to the manufacturer’s instructions.

### Library preparation

Libraries were prepared for Illumina sequencing using a two-step PCR protocol based on Gagnon et al. (2014) to assess mutation rates, and HDR efficiency and accuracy. The first PCR was performed using Q5 High-Fidelity DNA Polymerase (NEB) on genomic DNA to amplify a fragment that covered the targeted mutagenesis site. Successful amplification was verified by 1% agarose gel. The PCR products were diluted 1:4 and used as templates for a second PCR to barcode individual samples using primers containing adapters with indexes. Primers used for amplification of the target sites can be found in Supplementary File 1. Equal volumes of barcoded fragments were pooled to form a library, which was subsequently purified using the QiaQuick Gel Extraction Kit (Qiagen) according to the manufacturer's instructions. The library was sequenced on the MiSeq platform (Illumina) using MiSeq Kit v.3 with 300 bp paired-end reads.

### Mutation analysis

Preprocessing of the sequence data was done as previously described (Straume et al. 2021). Reads retained after filtering were mapped to the respective reference amplicon sequences using Muscle (v. 3.8.1551) (Edgar 2004). The processed sequence data was analyzed with custom Python scripts and visualized with Geneious Prime software (v. 11.0.12). Only variants with more than 100 reads were included. For deletion size analysis, only variants ≥ 1% were included. Total read counts and wild-type (WT) read counts were calculated for all samples. The WT counts were subtracted from the total read counts to find the total number of reads containing mutations. The numbers of different mutation-containing reads were subsequently used for calculating the integration rates.

### Statistics

The D’Agostino & Pearson Normality test was used to assess Gaussian distribution of the data. Since some groups did not follow a normal distribution, non-parametric tests were used. For the comparison between groups, we used the non-parametric Mann–Whitney or Wilcoxon paired tests. The tests were carried out using GraphPad Prism (v. 9.5.1).

## Results and discussion

### CRISPR/LbCas12a knock-out of *slc45a2*

**Fig. 1:**
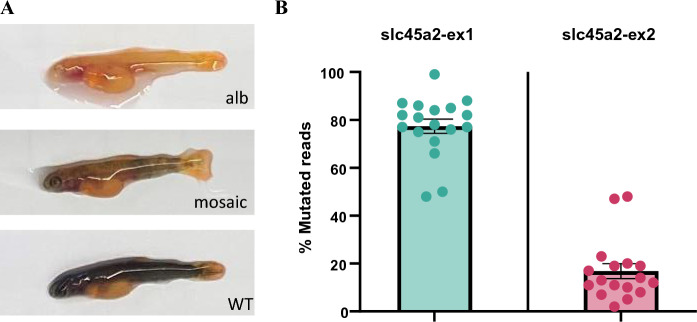
*slc45a2* knock-out using LbCas12a nuclease. **A** Albino (alb), mosaic, and wild-type (WT) phenotypes in salmon larvae injected with a LbCas12a RNP complex targeting the pigmentation gene *slc45a2*. **B** The degree of mutation in larvae was assessed using amplicon sequencing (MiSeq). The percentage (%) of reads supporting mutations is reported for crRNAs slc45a2-ex1 (n = 18) and slc45a2-ex2 (n = 17). Error bars indicate SEM

Atlantic salmon incubate at low temperatures and we therefore chose to use the LbCas12a nuclease due to AsCas12a showing less activity at 25 ℃ compared to LbCas12a in zebrafish (Moreno-Mateos et al. 2017), and no activity in rice (Hu et al. 2017). To test LbCas12a activity, we designed three different crRNAs, one targeting exon 1 and two targeting exon 2 of *slc45a2*. Two of the crRNAs (slc45a2-ex1 and slc45a2-ex2) were multiplexed, and one (slc45a2-ex2-2) was injected singly. In the group injected with a single RNP complex, no individuals exhibited an albino or mosaic pigmentation phenotype at the time of sampling, and we concluded that crRNA slc45a2-ex2-2 had no or very low efficiency. From the group injected with two RNP complexes, we sampled 19 larvae showing albino or mosaic pigmentation phenotypes (Fig. 1A; Supplementary File 2, Fig. S1). A non-injected control was included. Experimental data regarding the number of individuals injected, sampled, and exhibiting albino or mosaic pigmentation phenotypes can be found in Supplementary File 1.

HTS was used to assess the mutation rates in the individual samples and revealed varying efficiency of the two crRNAs (Fig. 1B; Supplementary File 3, Table S1). crRNA slc45a2-ex1 generated an average mutation rate of 77.3% (48.3–99.1%) whereas slc45a2-ex2 resulted in an average mutation rate of 16.8% (2.0–48.1%). In comparison, previous experiments using Cas9 mRNA and sgRNA produced similar mutation rates to the crRNA slc45a2-ex1 (Straume et al. 2020, 2021). However, editing efficiencies vary greatly between Cas9-experiments, also when using the same sgRNA. Various factors such as the Cas9 mRNA and sgRNA quality can affect the outcome. Furthermore, the egg quality, including survivability, and microinjection procedure may contribute to creating variation between experiments. The microinjection procedure involves aiming directly at the developing cell, which can be difficult due to the opaque nature of the eggs. The injection volume will also differ between individual embryos due to the opening of the needle. Taken together, our results show that LbCas12a is applicable as a tool for gene KO in Atlantic salmon, although with varying efficiency depending on the crRNA sequence, both within the same gene and even the same exon. Therefore, designing several crRNAs is recommended to ensure a high mutation rate and KO effect. This is especially important for salmon, where crossing out to F1 generation is impractical due to the long generation time.

### CRISPR/LbCas12a-mediated HDR knock-in of FLAG

**Fig. 2:**
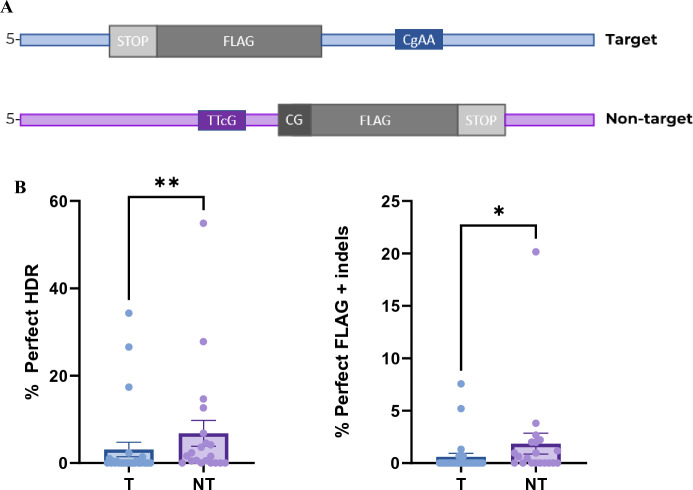
*slc45a2* FLAG knock-in using LbCas12a nuclease**. A** Asymmetrical ODNs containing 90/36 nt from each side of the LbCas12a cut sites, with a 27–29 nt insert comprised of the FLAG sequence followed by a stop codon (TAA). Additional nucleotides (CG) were added to keep the open reading frame for FLAG in the non-target strand ODN. PAM sites were mutated to avoid repeated cutting. **B** Salmon embryos injected with a LbCas12a RNP complex and either target (T, n = 27) or non-target (NT, n = 20) strand ODN template design. Integration rates were assessed by amplicon sequencing (MiSeq). Perfect HDR: sequence reads with perfect match to the template sequence. Perfect FLAG + indels: reads showing integration of FLAG but with indels on either side of the insert. Error bars indicate SEM. *indicate *P* < 0.05, **indicate *P* < 0.01

We used the slc45a2-ex1 crRNA in combination with a target or non-target strand FLAG ODN template (Fig. 2A) to investigate the possibilities of HDR-mediated KI using the LbCas12a nuclease. We sampled 27 individuals from the group injected with the target strand template and 20 individuals from the group injected with the non-target strand template (Supplementary File 2, Fig. S2 and S3).

The rate of perfect HDR occurring in individual larvae was assessed using amplicon sequencing data (Fig. 2B; Supplementary File 3, Table S2). Perfect HDR was defined as the perfect integration of the FLAG sequence, without indels in the insert itself, nor up- or downstream of the insert. When calculating the perfect HDR rate in each larva, we removed WT reads from the total reads obtained, ending up with the mutated reads. This allowed us to look at the amount of HDR events out of the total CRISPR events and removed potential variation between the two groups due to crRNA efficiency. Individual differences were observed; 9 out of 27 individuals in the group injected with the target strand template (T), and 14 out of 20 individuals in the non-target strand template (NT) group had sequence reads showing perfect HDR. Within these fish, the average percentage of perfect HDR was 9.4% (SEM 4.4%) for the T group and 9.7% (SEM 4.0%) for the NT group. Three fish from the T group and four fish from the NT group had perfect HDR above 10%: 17.4, 26.6, and 34.3% for T, and 12.7, 14.7, 27.8, and 54.9% for NT. Taken together, we achieved perfect HDR efficiencies similar to what we have previously seen with Cas9 mRNA in Atlantic salmon (Straume et al. 2021). We achieved high rates of perfect HDR using both target and non-target strand designs of the FLAG template. This contrasts with Moreno-Mateos et al. (2017) who observed almost no HDR using a target strand-oriented template, although with a small number of samples (Moreno-Mateos et al. 2017). On the other hand, and in agreement with Moreno-Mateos et al. (2017), our NT group gave significantly more perfect HDR than the T group.

For some sequence reads, the FLAG element had been correctly inserted but with indels up- or downstream of the insert (Fig. 2B; Supplementary File 3, Table S2). This occurred in 6 out of 27, and 11 out of 20 individuals in the T and NT group, respectively. The average percentage of reads displaying perfect FLAG + indels was 2.7% (SEM 1.2%) in the T group, and 3.4% (SEM 1.7%) in the NT group. We have previously demonstrated that template polarity determines the location of indels when doing KI with Cas9 and symmetrical ODNs in salmon (Straume et al. 2020). Later, we found that the indel location was determined by template polarity also when using asymmetrical ODNs for the *dnd* gene, but not for *slc45a2* (Straume et al. 2021). In the current study, we had few samples displaying perfect FLAG + indels, but observed a trend in ODN polarity-driven indel positioning also for LbCas12a (Supplementary File 3, Table S2).

Furthermore, we observed sequence reads where HDR had occurred with several errors, such as partially inserted FLAG or FLAG containing substitutions and insertions. Imperfect HDR reads were found in 19 out of 27 individuals in the T group, and in 15 out of 20 individuals in the NT group. The average of imperfect HDR reads in these fish were 2.9% (SEM 0.9%) and 6.0% (SEM 1.5%) in the T and NT group, respectively (Supplementary File 3, Table S2).

### Combining LbCas12a and Cas9

The possibility of using Cas12a and Cas9 at the same time could be practical both for KO and KI approaches. We microinjected salmon embryos with LbCas12a/slc45a2-ex1 RNP and non-target strand FLAG template in combination with Cas9/slc45a2-ex6 RNP and target strand FLAG template. The FLAG sequences were in the same orientation to prevent hybridization between the two different ODNs. A total of 29 individuals were sampled (Supplementary File 2, Fig. S4).

**Fig. 3:**
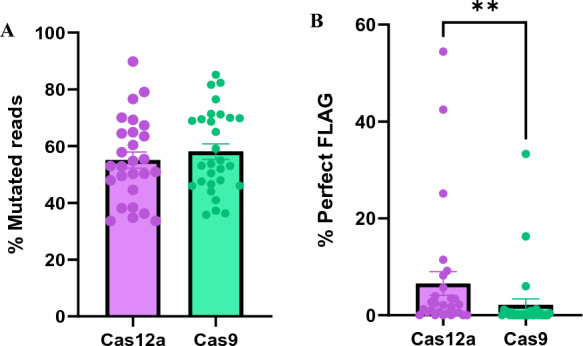
slc45a2 knock-out (**A**) and FLAG knock-in (**B**) using LbCas12a and Cas9 nucleases. Salmon embryos were co-injected with LbCas12a and Cas9 RNPstargeting exons 1 and 6 of the pigment gene *slc45a2*, respectively, and FLAG templates. **A** The mutation rates were assessed by sequencing of the target sites. The percentage (%) of reads supporting mutations is reported for LbCas12a (n = 27) and Cas9 (n = 29) nucleases. **B** The rates of perfect FLAG integration (%) calculated by dividing the perfect FLAG reads by the total number of mutated reads. Error bars indicate SEM. **indicate *P* < 0.01

In addition to testing LbCas12a RNP for the first time in Atlantic salmon, this is also the first time we report using Cas9 RNP. Because the two RNPs were combined, we assume equivalent amounts of the RNPs were injected in each embryo, removing uncertainty regarding the delivery. When comparing LbCas12a and Cas9, we saw that the two nucleases produced similar mutation rates for the crRNA and sgRNA tested in this study, with an average of 55.1% for LbCas12a/slc45a2-ex1, and 58.1% for Cas9/slc45a2-ex6 (Fig. 3A; Supplementary File 3, Table S3). Resembling mutation efficiencies agrees with what has been reported previously in zebrafish (Meshalkina et al. 2020). Furthermore, the number of indel variants generated by the nucleases was also found to be similar (Supplementary File 2, Fig. S5). GE is commonly used to generate KO animals in order to study gene function. Here, achieving a large deletion is preferred as it increases the chance of loss-of-function even if the indels lead to in-frame mutations. Previous studies in rice and zebrafish have reported that LbCas12a generates larger deletions compared to Cas9 (Hu et al. 2017; Meshalkina et al. 2020). As the target sites for our crRNA and sgRNA are located in different exons of *slc45a2*, we cannot compare the two nucleases directly. However, in our data we observed that an average of 63.4% of the LbCas12a/slc45a2-ex1-generated deletions were 10 bp or more, whereas an average of 29.4% of the Cas9/slc45a2-ex6-induced deletions were 10 bp or more (Supplementary File 3, Table S4)*.* Nevertheless, as we have only acquired deletion size data from one target site for LbCas12a and one for Cas9, we cannot conclude whether this outcome is attributed to the editing features of LbCas12a, or other factors such as the specific target sites or initiation of the microhomology-mediated end joining repair pathway. It is also worth noting that since the RNPs were multiplexed, they might influence each other. For example, we observed complete removal of the region between the cut sites of the two nucleases. To examine the extent of this large deletion, we performed PCR using primers targeting upstream of the LbCas12a cut site and downstream of the Cas9 cut site. In 8 out of 28 samples, we observed a gel band indicating that the entire region of 30 kb was removed for an unknown proportion of the CRISPR events (Supplementary File 2, Fig. S6). While certain GE approaches support the generation of large deletions, the target sites should be carefully considered when multiplexing crRNAs and sgRNAs.

As for the HDR events in the co-injected individuals, the sequencing revealed that perfect integration of FLAG (regardless of indels up- or downstream of the insert) occurred significantly more often at the LbCas12a cut site compared to the Cas9 cut site (Fig. 3B; Supplementary File 3, Table S3). At the LbCas12a cut site, 22 out of 28 samples had perfect FLAG integration. Within these samples, the average was 8.3% (SEM 3.0%) and the highest individual displayed 54.4% perfect FLAG. In contrast, we observed perfect FLAG integration in 11 out of 29 samples at the Cas9 cut site, with an average of 5.6% (SEM 3.1%) and the highest individual having 33.3% perfect FLAG reads. Higher HDR efficiency using LbCas12a compared to Cas9 has previously been demonstrated in zebrafish, where LbCas12a in combination with the optimal DNA donor was found to improve HDR in two of four loci tested when compared to SpCas9 (Moreno-Mateos et al. 2017). However, our data includes FLAG integration only at a single locus using crRNA and sgRNA targeting different exons. Further studies are necessary to corroborate whether LbCas12a exhibits improved HDR efficiency compared to Cas9 in Atlantic salmon.

Surprisingly, exon 1 fragments were found at the cut site of Cas9 in exon 6, and vice versa, exon 6 fragments at the cut site of LbCas12a in exon 1. Exon 1 fragments, likely originating from the homology arms of the LbCas12a-associated template, had been inserted into the Cas9 cut site to a greater extent than the other way around. In 20 out of 29 fish, an average of 4.7% (SEM 1.5%) of the reads showed that exon 1 fragments had been inserted into the Cas9 cut site. On the other hand, in 3 out of 27 fish, an average of 2.3% (SEM 1.0%) of the reads showed exon 6 fragments at the LbCas12a cut site (Supplementary File 3, Table S5).

## Conclusion

In the present work, we successfully applied the LbCas12a nuclease to KO the *slc45a2* gene in Atlantic salmon. Application of this nuclease brings important advantages such as additional target opportunities, especially in AT-rich regions of the genome, owing to the 5′-TTTV-3′ PAM requirement. Furthermore, the deletions created by LbCas12a were larger than the Cas9-mediated deletions for the crRNA and sgRNA tested in this study, which is likely to be an advantage for gene KO studies. We also performed KI of a FLAG sequence element by using ODN templates together with the LbCas12a RNP, achieving perfect HDR rates of up to 54.8% in individual larva. Finally, when comparing larvae injected simultaneously with both LbCas12a and Cas9 RNPs, we observed similar mutation rates, but more FLAG integration at the LbCas12a cut site, suggesting improved KI using the LbCas12a nuclease. However, as we only targeted a single gene using one crRNA and one sgRNA, further studies are necessary to generate more data on the KI possibilities of LbCas12a in salmon. Establishing the use of CRISPR/LbCas12a expands the toolbox for GE in salmon and may also inspire the use of this nuclease in other non-model species.

### Supplementary Information

Below is the link to the electronic supplementary material.

Supplementary file1 (XLSX 17 KB)

Supplementary file2 (DOCX 3843 KB)

Supplementary file3 (XLSX 39 KB)

## Data Availability

All data used for calculating the different rates can be found in Supplementary File 3.
