# Supplementary material for: In vivo CRISPR/LbCas12a-mediated knock-in and knock-out in Atlantic salmon (Salmo salar L.)
Source: Transgenic Res. 2023 Sep 21;32(6):513–21. doi: 10.1007/s11248-023-00368-4 (PMC10713776; doi:10.1007/s11248-023-00368-4)
Supplement: Supplementary file 2 — Supplementary file2 (DOCX 3843 KB) [file 11248_2023_368_MOESM2_ESM.docx]

Supplementary File 2: Figures

| *In vivo* CRISPR/LbCas12a-mediated knock-in and knock-out in Atlantic salmon (*Salmo salar* L.) |
| --- |
|  |
| Mari Raudstein^1^, Erik Kjærner-Semb^1^, Morten Barvik^1^, Silje Broll^1^, Anne Hege Straume^1^, Rolf B. Edvardsen^1^ |


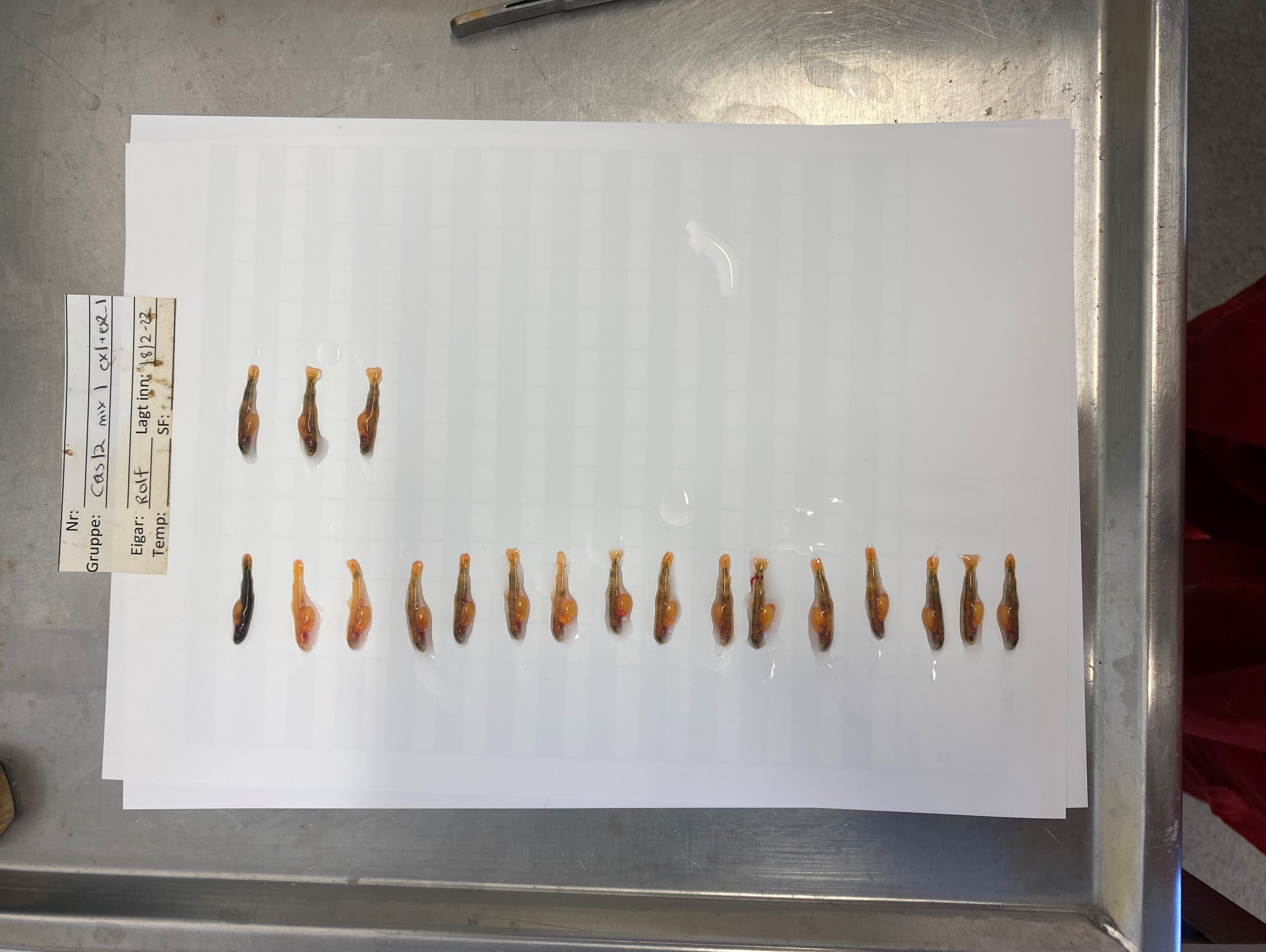


WT

**Supplementary Fig. S1** Sampling of salmon larvae injected with LbCas12a RNP targeting exon 1 and exon 2 of slc45a2. A total of 19 larvae showing albino or mosaic pigmentation phenotype were sampled (one additional larvae was sampled after the photo was taken). One pigmented (labelled WT) larvae for comparison


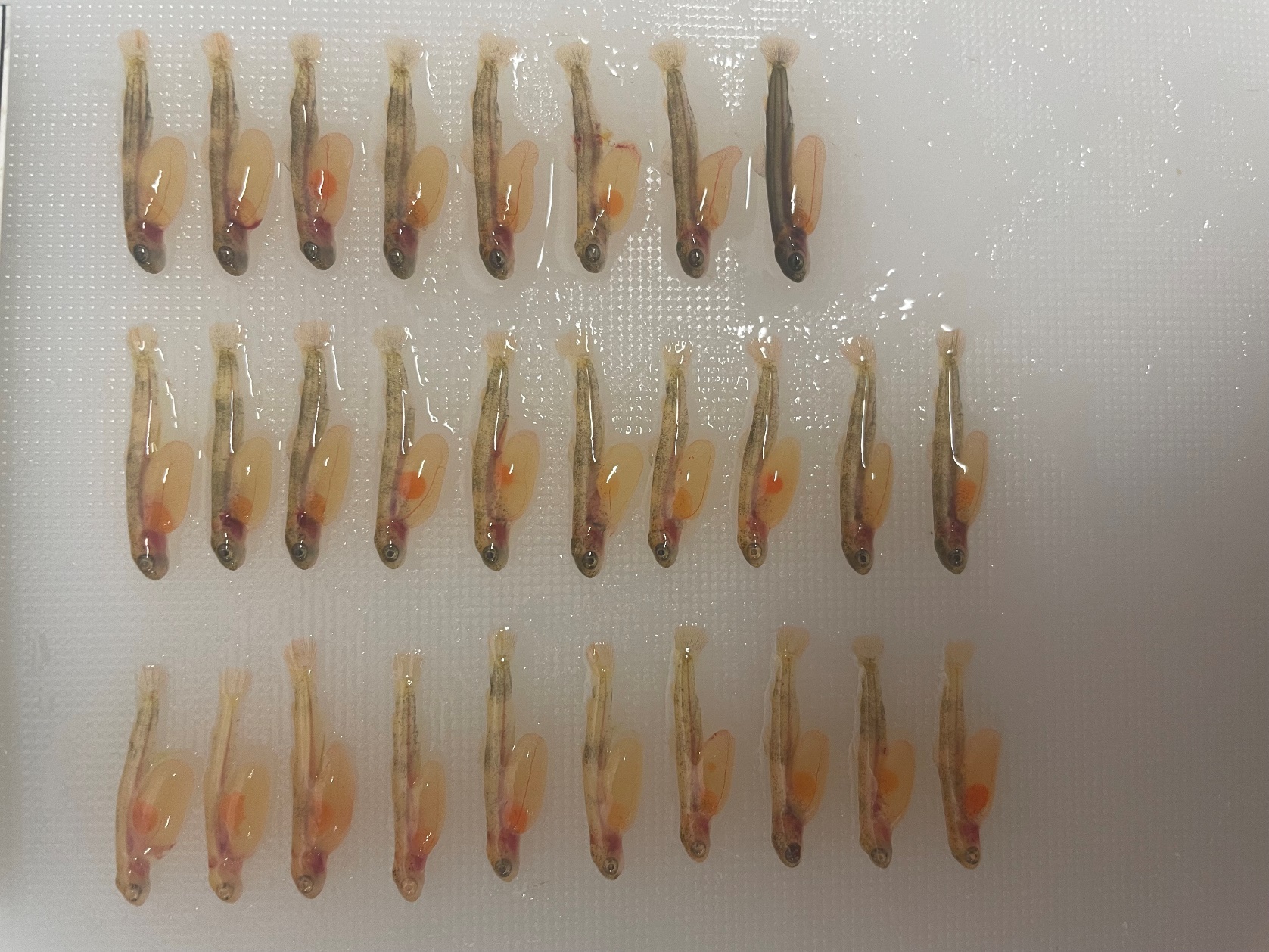


WT

**Supplementary Fig. S2** Sampling of salmon larvae injected with LbCas12a RNP targeting exon 1 of slc45a2 in combination with target strand ODN template. A total of 27 individuals showing albino or mosaic pigmentation phenotypes were sampled. One wild-type (labelled WT) larvae for comparison


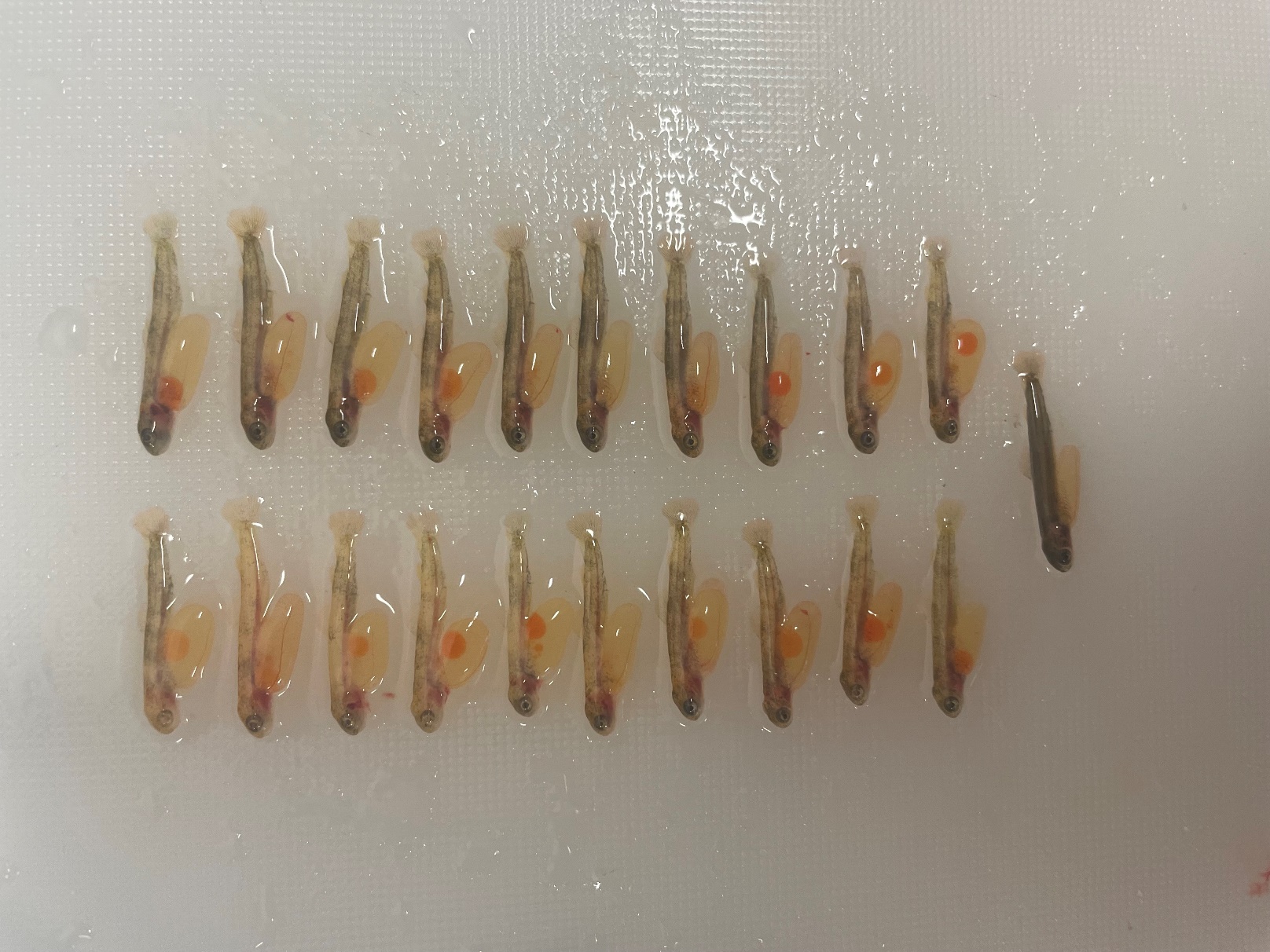


WT

**Supplementary Fig. S3** Sampling of larvae injected with LbCas12a RNP targeting exon 1 of slc45a2 in combination with non-target template. A total of 20 individuals showing albino or mosaic pigmentation phenotypes were sampled. One wild-type (labelled WT) larvae for comparison


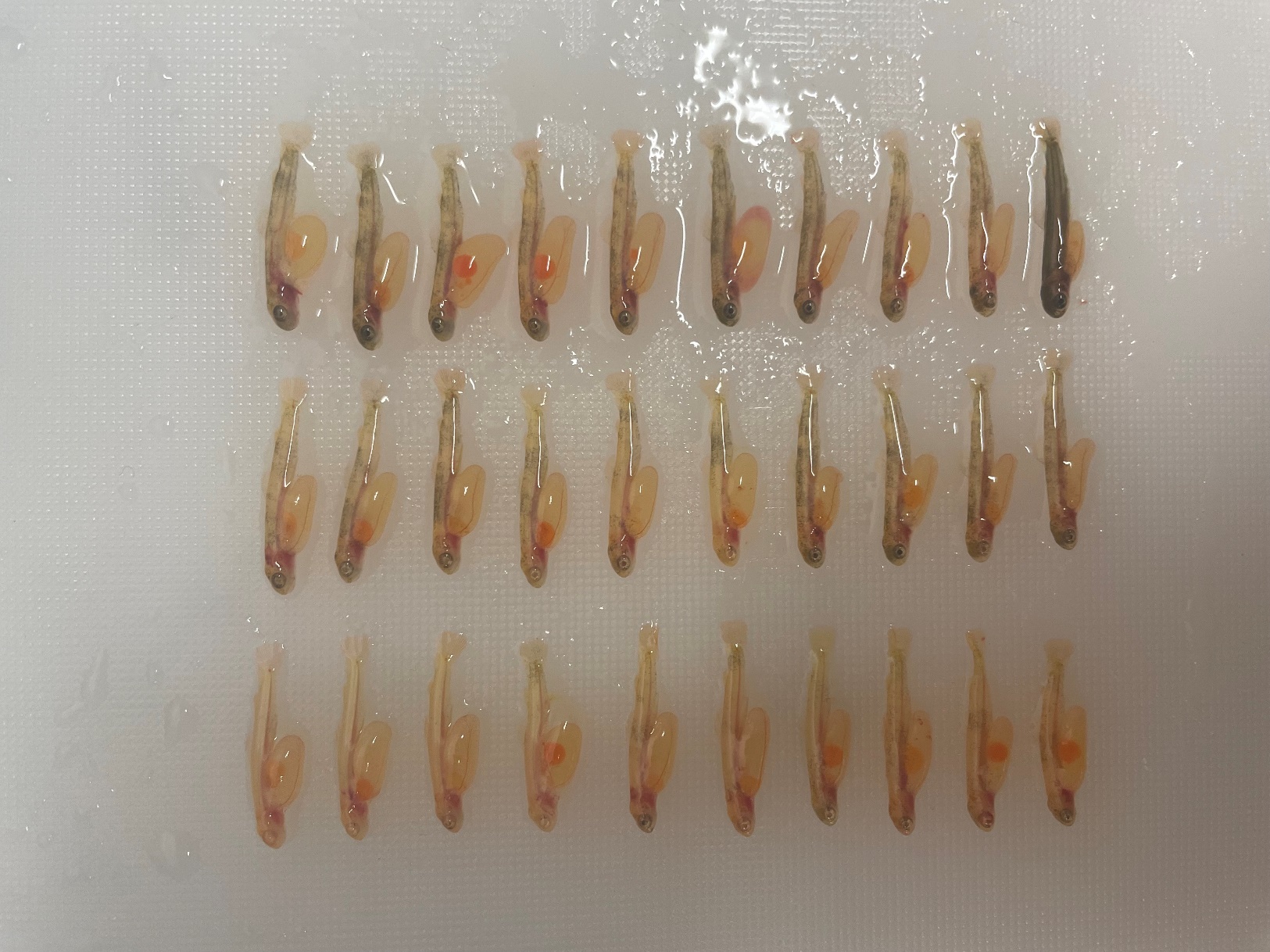


WT

**Supplementary Fig. S4** Sampling of larvae injected with LbCas12a RNP targeting exon 1 of slc45a2 and non-target template in combination with Cas9 RNP targeting exon 6 of slc45a2 and target template. A total of 29 individuals showing albino or mosaic pigmentation phenotypes were sampled. One wild-type (labelled WT) larvae for comparison

**Supplementary Fig. S5** Number of indel variants in each individual larvae injected with LbCas12a RNP and non-target strand template, in combination with Cas9 RNP and target strand template


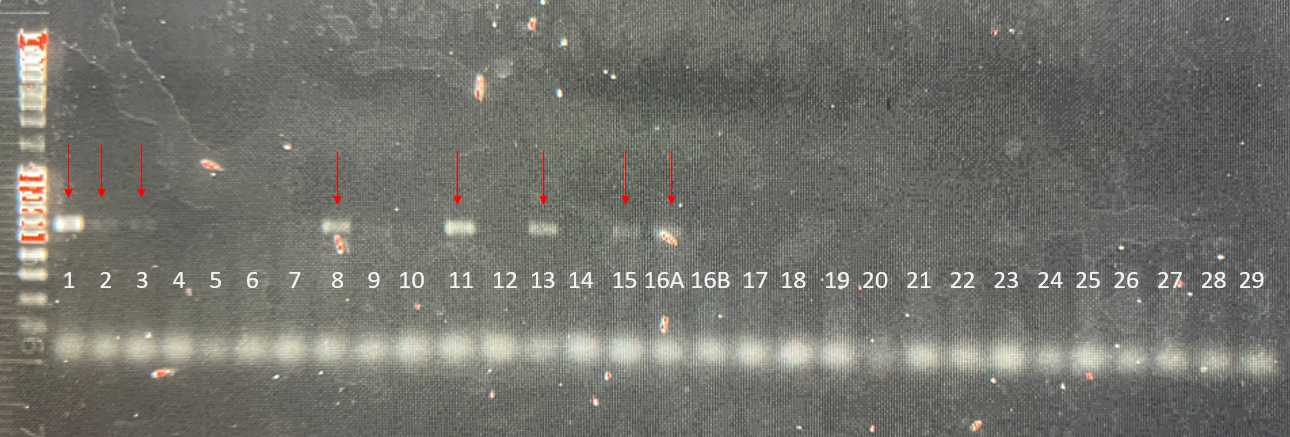

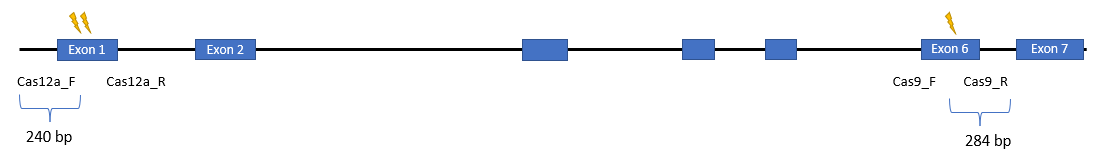


600 bp

**Supplementary Fig. S6** Knock-out (KO) of a 30 kb region in slc45a2 using LbCas12a and Cas9. Salmon embryos were injected with two RNPs: LbCas12a and Cas9 targeting exon 1 and exon 6 of slc45a2, respectively. Gel electrophoresis of fragments amplified using Cas12a F primer and Cas9 R primer was performed to determine excision of the whole region between the cut sites of the two nucleases. The red arrows indicate samples where the whole region has been KO. The 240 and 284 bp labels illustrate the number of bases from the primer binding to the cut site, meaning that the resulting band should be 524 bp. Both primers have adapters of 30 bp length, so the total band size is 584 bp
